# Combining Infrared Thermography with Computer Vision Towards Automatic Detection and Localization of Air Leaks

**DOI:** 10.3390/s25113272

**Published:** 2025-05-22

**Authors:** Ângela Semitela, João Silva, André F. Girão, Samuel Verdasca, Rita Futre, Nuno Lau, José P. Santos, António Completo

**Affiliations:** 1 Centre of Mechanical Technology and Automation (TEMA), Department of Mechanical Engineering, University of Aveiro, 3810-193 Aveiro, Portugal; jpls@ua.pt (J.S.); andrefgirao@ua.pt (A.F.G.); samuelcverdasca@ua.pt (S.V.); jps@ua.pt (J.P.S.); 2 Intelligent Systems Associate Laboratory (LASI), 4800-058 Guimarães, Portugal; nunolau@ua.pt; 3 Bosch Thermotechnology, S.A., Cacia, 3800-627 Aveiro, Portugal; 4 Institute of Electronics and Informatics Engineering of Aveiro (IEETA), Department of Electronics, Telecommunications and Informatics, University of Aveiro, 3810-193 Aveiro, Portugal

**Keywords:** air leaks, infrared thermography, computer vision, algorithm, localization, leak aperture, distance

## Abstract

This paper proposes an automated system integrating infrared thermography (IRT) and computer vision for air leak detection and localization in end-of-line (EOL) testing stations. This system consists of (1) a leak tester for detection and quantification of leaks, (2) an infrared camera for real-time thermal image acquisition; and (3) an algorithm for automatic leak localization. The python-based algorithm acquires thermal frames from the camera’s streaming video, identifies potential leak regions by selecting a region of interest, mitigates environmental interferences via image processing, and pinpoints leaks by employing pixel intensity thresholding. A closed circuit with an embedded leak system simulated relevant leakage scenarios, varying leak apertures (ranging from 0.25 to 3 mm), and camera–leak system distances (0.2 and 1 m). Results confirmed that (1) the leak tester effectively detected and quantified leaks, with larger apertures generating higher leak rates; (2) the IRT performance was highly dependent on leak aperture and camera–leak system distance, confirming that shorter distances improve localization accuracy; and (3) the algorithm localized all leaks in both lab and industrial environments, regardless of the camera–leak system distance, mostly achieving accuracies higher than 0.7. Overall, the combined system demonstrated great potential for long-term implementation in EOL leakage stations in the manufacturing sector, offering an effective and cost-effective alternative for manual inspections.

## 1. Introduction

End-of-line (EOL) quality assessment is a critical step in the industrial manufacturing process, since it ensures that manufactured products meet the required functionality and safety specifications before packaging and shipping to customers or distribution centers [1]. Pressure differential with a reference volume has been by far the most widely used technique for leak detection in EOL leakage stations due to its reliability and sensitivity [2,3]. Given the current necessity to use fuel and natural resources wastefully in EOL leakage tests, several industrial sectors are trying to adopt more environmentally friendly strategies to test their products [4,5]. In this instance, air has been exploited as an alternative medium in EOL leakage tests, since it can (1) provide faster leak detections; (2) maintain the product dry and clean after the test; and (3) reduce the waste and potential pollution associated with fuel and natural resources [5]. However, the use of air as the medium to detect leaks might hamper their visual localization. Thus, a manual inspection is often required to accurately locate each leak, generating significant delays in the production line and an overall reduction of productivity [6]. To overcome this bottleneck, it is necessary to explore new technologies with the capability to automatically and intuitively localize air leaks. Additionally, the versatility of such systems must enable feasible implementation in EOL leakage testing stations in a simple and cost-effective manner.

From an industrial perspective, infrared (IR) thermography (IRT) provides several advantages, such as (1) high precision and sensitivity; (2) fast or real-time response; (3) being easy and safe to operate; and (4) low power consumption [7,8,9]. In an air leak scenario, the expansion of the pressurized air generates a significant temperature reduction that can be easily detected with IRT devices in the form of thermal images that can be used directly or further processed to accurately determine the location of the leak [10,11]. Vision-based technologies, usually employed on industrial inspection systems [12,13,14], can be easily used for the analysis of these thermal images to measure the temperature differences between the leaked gas and the surrounding environment and pinpoint the leak location [15]. However, most of the reported vision technologies are excessively complex, hindering their industrial implementation in a cost-efficient manner, or are inefficient in air leak localization [15]. Given these considerations, this paper reports the combination of IRT with computer vision to develop a leak detection and localization system for implementation on an EOL leakage testing station, consisting of three main components: (1) A typical universal leak tester for air leak detection and quantification, (2) an IR camera for IR image acquisition; and (3) an algorithm for air leak localization. At this point, it is hypothesized that the combination of these three components will facilitate air leak detection, quantification, and localization, providing an effective quality control tool for the industrial manufacturing sector.

## 2. Theoretical Background

### 2.1. Principles of IRT

All objects with temperatures above absolute zero emit electromagnetic radiation in the IR region [16]. IRT devices, equipped with specialized cameras and interchangeable optics, detect this radiation by converting the captured energy into an electronic video signal. This signal is then processed into a visible image, where different colors (or grayscale levels) represent varying energy intensities [17]. It should be noted that while the emitted energy is proportional to the surface temperature, the energy that is actually detected by the IRT device is highly dependent on the emissivity coefficient of the object’s surface and the surrounding environment [18,19]. Emissivity, which defines an object’s ability to emit IR energy, ranges from 0 and 1, with 0 attributed to low-emissivity materials (polished metals) and 1 to high-emissivity materials (polymers) [7]. Besides the temperature of the object itself, the temperature of the background radiation of the surrounding environment can also have a significant influence on the performance of IRT devices. For instance, if the background has a similar temperature to the target object, the reduced contrast can hamper object detection [11].

In the context of gas leak detection, the leaked gas, typically at a lower temperature than its surroundings, can absorb a portion of the IR radiation emitted by the background, altering the spectrum of radiation received by the IRT device [11]. Therefore, a region with lower intensity appears in the thermal image that can be attributed to the presence of leaked gas [18].

### 2.2. IRT-Based Leak Detection Systems

IRT has been extensively exploited for the development of leak detection systems, using both active and passive approaches. Active IRT systems typically combine an IR camera with an external radiation source, enabling enhanced leak detection capabilities [8]. Kasai et al. proposed the use of a carbon IR emitter to enhance the real-time visualization of propane leaks from sampling bags placed at varying distances (1, 1.75, and 2.5 m) [20]. Exploring this concept further, Barber et al. integrated a similar active-based imaging system (employing a silicon nitride emitter instead) onto a mobile robotic platform, which autonomously moved across a predefined path to perform remote CO_2_ sensing [21]. While promising, the implementation of these active IRT methodologies for leak detection might not be viable in industrial contexts due to their high costs and implementation complexity.

Alternatively, passive IRT systems offer more practical applications, relying solely on background thermal radiation [22]. Bach et al. employed an IR camera for water leak localization in small and buried pipelines. Out of 27 leak sites, the camera successfully identified 59% of leaks, with 22% yielding inconclusive results due to environmental interference such as solar radiation, rainfall, and humidity [23]. Adefila et al. employed a similar IR camera for the detection of CO_2_ leaks in controlled laboratory conditions, using varying pressures (100, 150, 200, 250, and 300 kPa) and leak apertures (0.15, 0.3, 0.5, 1, 3, and 5 mm). The results confirmed these IR cameras provided accurate temperature readings, with a maximum temperature differential of 1.6 °C, demonstrating their acceptable detection capabilities under controlled conditions [10].

In an effort to facilitate and automatize leak detection, it has been reported the implementation of computer vision-based processing algorithms within leak detection systems. Indeed, Xie et al. combined IRT with Faster Region-based Convolutional Neural Networks for water leak localization in visually complex environments. This system proved highly effective across varied lighting conditions, viewing angles, and disturbances, consistently achieving mean average precision (mAP) values above 0.98 [24]. Similarly, Xu et al. proposed a gas leak detection system for leak detection and segmentation comprising a cooled mid-wave IR camera and a YOLO-v8-based network, with a global attention mechanism to improve feature extraction. The results validated the superior performance of this system both in terms of gas leak detection and segmentation of gas plumes, achieving mAPs of 0.95 [25]. Ma et al. developed an active leak detection system featuring (1) background enhancement devices; (2) external thermal pulse excitation; and (3) a machine learning-based model for leak recognition. The results demonstrated that this system enhanced the contrast between the leaks and the background, enabling the model to achieve accuracies over 0.95 for leak identification [26].

Despite their success, the reported advanced computer vision-based approaches introduce higher computational complexity that might (1) significantly reduce the cost-effectiveness of the leak detection system; (2) be incompatible with existing hardware in an industrial setting; and (3) require additional specialized personnel for network implementation and maintenance. To address these limitations, simpler and more manageable algorithms have been alternatively proposed. For instance, Jadin et al. developed a lightweight vision-based algorithm that processed the acquired IR thermal images to enhance and segment regions of interest and perform feature extraction and classification of leak scenarios. The results showed that the developed vision-based system was able to correctly indicate whether a gas leak was present with 95 and 96% of accuracy for non-leak and leak scenarios, respectively [27]. Similarly, Zhao et al. proposed a leak detection model comprising (1) an image-enhancement algorithm based on Gaussian filtering and adaptive histogram segmentation to improve the quality of the thermal images and (2) a gas-leak area detection algorithm based on visual background extractor for further leak feature extraction. Tested under varying environmental conditions (indoor and outdoor) and gas types (sulfur hexafluoride and carbon tetrafluoride), this model achieved F1-scores above 0.85 in most scenarios [28]. Besides leak detection, these vision-based algorithms have also been adapted for leak quantification. Indeed, Hassan et al. employed a similar image processing algorithm to estimate the leak aperture in thermal images, to obtain an approximation of the leak flow rates [29].

Despite these advances, most of the current research has been focused on leak detection in large pipelines, with sizable and optimally exposed leaks. However, their performance might not be easily translated to smaller industrial settings such as the EOL leakage testing station, where the circuits possess smaller dimensions and the tubes are intertwined and overlapped over each other, increasing the difficulty of leak visualization. In this regard, the present study aims to address these limitations by proposing a solution suited to the constraints and dimensions of EOL testing environments.

## 3. Proposed Methodology

### 3.1. Thermal Image Acquisition Setup

**Figure 1:**
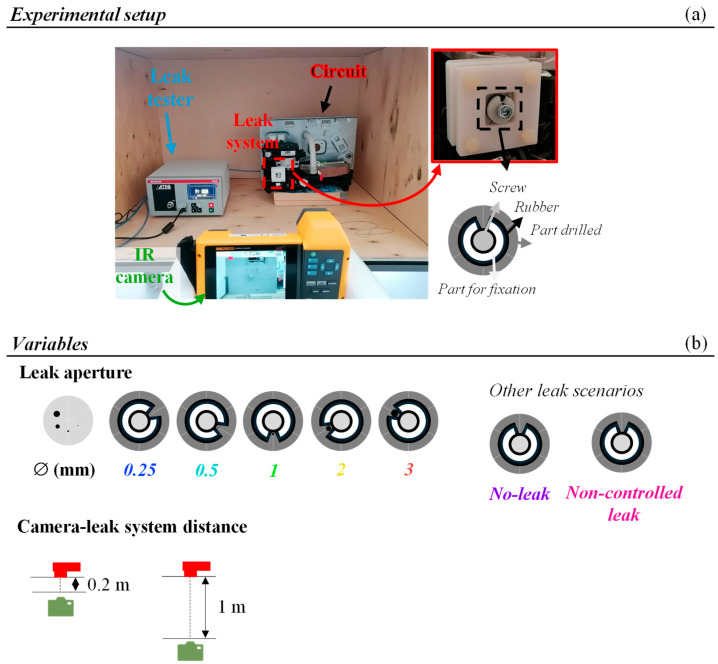
Experimental setup for leak localization comprising an IR camera, a universal leak tester, and a circuit with an embedded leak system (**a**) and schematic representation of the different variables evaluated: 0, 0.25, 0.5, 1, 2, and 3 mm leak apertures and distances of 0.2 and 1 m between the IR camera (in green) and the circuit with the embedded leak system (in red) (**b**).

The experimental setup employed for thermal image acquisition, shown in Figure 1a, consisted of (1) a universal leak tester ATEQ F620 using differential sensor pressure testing for evaluation of each circuit’s airtightness (ATEQ, Les Clayes-sous-Bois, France); (2) an IR camera Fluke TiX580 with a thermal sensitivity of ≤0.05 °C at 30 °C and spatial resolution of 0.93 mRad (Fluke Corporation, Everett, WA, USA); and (3) a closed hydraulic circuit (provided by Bosch Thermotechnology, S.A., Cacia, Portugal) with an embedded leak system designed to provide different leak apertures. Both the IR camera and the ATEQ were placed in a wood box, as depicted in Figure 1a, to reduce the interference of the environmental lighting. Different variables were evaluated (Figure 1b); namely, five different leak apertures (0.25, 0.5, 1, 2, and 3 mm) and two camera–leak system distances (0.2 and 1 m). It should be emphasized that these distances were selected to contemplate a wide range of distances encountered in EOL testing stations.

For each leak aperture, the fixation part and the rubber were simultaneously rotated to expose each aperture and tightened with a screw to guarantee air flow from the exposed aperture only. It should be noted that besides the referred leak apertures, two additional scenarios were evaluated: (1) no-leak (0 mm), where the fixation part and the rubber were rotated to expose an area with no leak aperture; and (2) non-controlled leak (NC-leak), where the screw was slightly unscrewed to generate considerable air flow.

Additionally, a preliminary test was performed in an industrial setting (Bosch Thermotechnology, S.A.), where the environmental lighting conditions were not controlled, using an identical leak tester and hydraulic system with a simulated air leak. In this case, the leaks were triggered by disassembling components of the circuit to generate a large leak. The distance between this hydraulic circuit and the IR camera was around 1 m.

### 3.2. Leakage Experiments

Compressed air was supplied to the ATEQ at 2.5 bar. All leakage experiments were conducted during the airtightness test of the universal leak tester. The equipment considered a leak when the flow of air was higher than 0.025 Pa·m^3^/s. This leak rate was provided by Bosch Thermotechnology as the reference value, which was determined experimentally to comply with safety regulations. Prior to each set of experiments, the IR camera’s emissivity setting was standardized to a fixed value of 0.95. Furthermore, the temperature was periodically and automatically calibrated, as well as the brightness and contrast of the camera. For each leak scenario (*n* = 3), the thermal and visible recordings captured using the IR camera were either stored and later processed or directly fed to the leak localization algorithm.

Data from the universal leak tester, namely the air pressure and airtightness test time, were also used to calculate the air leak rates (*n* = 5) using the following Equation (1).

(1) $$Leak\;Rate=\frac{Air\;pressure\;\left(Pa\right)\times Volume\;of\;air\;within\;the\;circuit\;(m^{3})}{Airtightness\;test\;time\;(s)}$$

### 3.3. Algorithm for Automatic Leak Localization

To enable real-time automatic localization of air leaks using the IR camera, a Python-based algorithm (version 3.9.0.) was developed to interface directly with the camera and obtain the captured IR video in real-time. This algorithm applied fundamental principles of computer vision and image processing to the collected frames, using the OpenCV library, to locate potential leaks.

**Figure 2:**
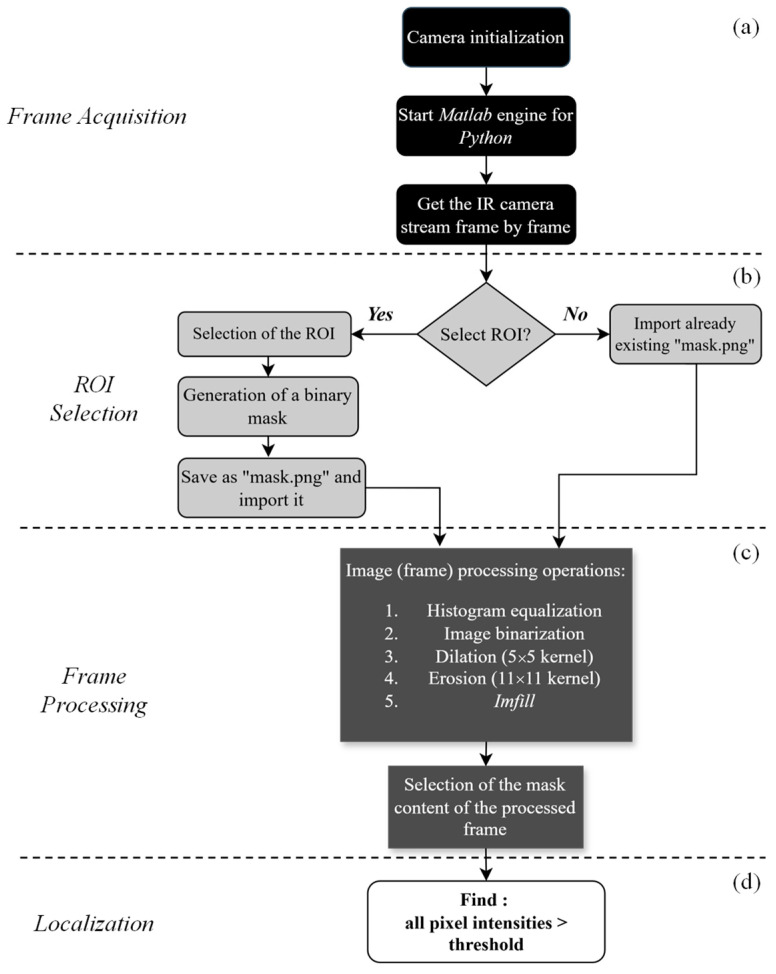
Python script for air leak localization: frame acquisition (**a**), ROI selection (**b**), frame processing (**c**), and localization (**d**).

A MATLAB (version R2023b) toolkit, specifically designed for Fluke devices, provided a set of functions that enabled direct access to the IR camera’s live feed. To bridge the gap between MATLAB and Python, the MATLAB engine for Python was employed, allowing seamless execution of MATLAB commands within the Python script. This integration enabled full automation of the leak localization process, from camera initialization to real-time leak detection and localization (Figure 2).

Once the camera was powered on and properly connected to the computer, the MATLAB engine was launched via Python. During this process, the necessary dynamic link library files were loaded from Fluke and the serial number of the connected IR camera was retrieved to establish the video stream (Figure 2a). In each iteration, two matrices were generated: (1) an image matrix representing the current frame and (2) a temperature matrix containing the corresponding temperature values for each pixel. The image data were then converted into a NumPy array and transformed into a grayscale representation in Python for further processing.

**Figure 3:**
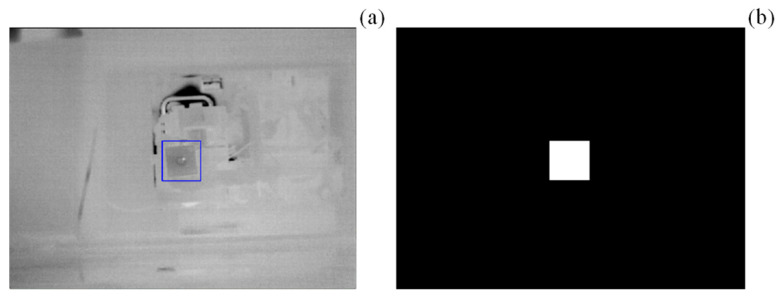
Manually defined ROI (in blue) (**a**) and the corresponding mask/binary image obtained (**b**).

To enhance the accuracy of leak detection, a Region of Interest (ROI) was defined within the image (Figure 2b). Given potential variations between experiments due to automatic camera color calibration, a manual method was initially implemented for ROI definition. The algorithm first scanned the system to check for the presence of a mask.png file, which contains a predefined binary image restricting processing to the designated ROI (Figure 3). If a valid mask.png file was detected, the user had the option to either use the existing ROI or define a new one. To ensure consistency across different processing stages, all frames and masks were resized to a standardized resolution of 640 × 480 pixels, which aligns with the native resolution of the IR camera.

**Figure 4:**
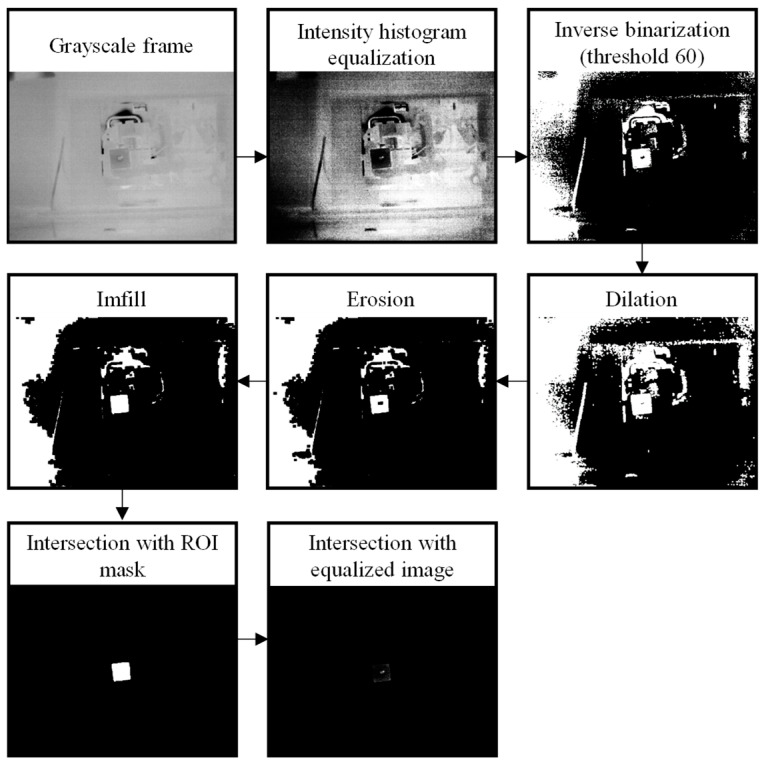
Frame processing steps; namely, the intensity histogram equalization, inverse binarization, dilation, erosion, imfill, and intersection with ROI mask and equalized image.

The selected grayscale frames were then processed (Figure 2c), employing a series of steps depicted in Figure 4. First, an intensity histogram equalization technique was implemented to address the inconsistencies in the lighting conditions. This technique normalized the distribution of the pixel’s intensities across the image, uniformizing the brightness and contrast between frames. Afterwards, an inverse binarization process was performed with a threshold of 60 to obtain (in white) the specific region where the leaks are. The resulting image underwent dilation and erosion using 5 × 5 and 11 × 11 kernels, respectively, after which a custom function—imfill—was employed to fill the white areas within the ROI. Two intersections were then performed consecutively to isolate the specific region: (1) The intersection of the processed frame with the initially imported mask and (2) the intersection of the intersected image with the original equalized image.

**Figure 5:**
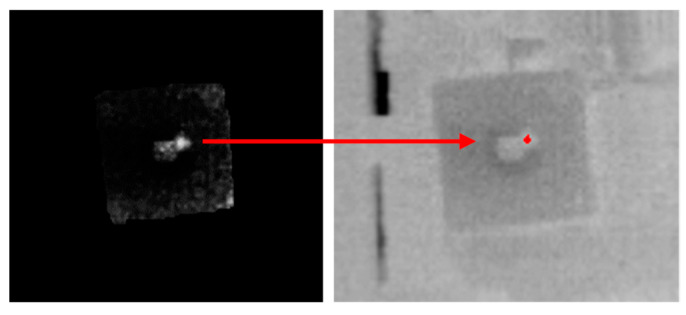
Localization and labeling of the areas (red dot) with pixel intensities above the defined threshold.

The final operation of the algorithm was the localization of the air leaks (Figure 2d). Given that areas with lighter colors were associated with lower temperatures, the algorithm was designed to identify these specific regions by defining a threshold value between 0 and 255. The values 120, 150, 172, and 190 were evaluated as thresholds to determine their effectiveness in isolating the whiter regions associated with air leaks. These values were selected through an iterative trial-and-error process, based on the visual clarity of the detected leaks in different testing conditions. The algorithm then marked the pixels with intensity above the referred threshold in red on the original frame (Figure 5).

#### Performance Evaluation

To assess the algorithm’s performance, several performance evaluation metrics were implemented. In this instance, both the total number of frames of the IR videos and the frames with visible leaks were used to assess the number of true positive (TP), true negative (TN), false positive (FP), and false negative (FN) results. These values were calculated by applying the following steps: (1) For each video, the range of frames corresponding to the presence of a leak within the ROI was determined, as well as the specific area (as a box) of the ROI where the leak was found; (2) each video was fed to the algorithm for detection; and (3) frame-by-frame, the corresponding variable was incremented if the following conditions were satisfied: TP, if the algorithm found a leak in the true detection frame and in the true detection area; FN, if the algorithm could not find a leak in the true detection frame and in the true detection area; FP, if the algorithm found a leak in the true detection frame and outside of the true detection area; and TN, if the algorithm could not find a detection outside the true detection frame range. These values allowed the calculation of the following metrics: (i) *Recall*, indicating the fraction of leaks correctly identified (TPs) (2); (ii) *Precision*, denoting, from all identified leaks, the fraction that was correctly identified (TPs) (3); (iii) *Specificity*, corresponding to the algorithms ability to correctly identify TNs (4); and (iv) *Accuracy*, indicating how often the algorithm correctly identified leaks (TPs) and no-leaks (TNs) (5).

(2) $$Recall=\frac{TP}{TP+FN}$$

(3) $$Precision=\frac{TP}{TP+FN}$$

(4) $$Specificity=\frac{TN}{FP+TN}$$

(5) $$Accuracy=\frac{TP+TN}{TP+FP+FN+TN}$$

### 3.4. Statistical Analysis

Statistical significance was determined by performing one-way analysis of variance (ANOVA) followed by post hoc Tukey’s test. Significance was accepted at *p*-values below 0.05.

## 4. Results and Discussion

In this work, an automatic leak detection and localization system was designed for possible implementation in an EOL leakage testing station by combining IRT and computer vision techniques. Its performance was assessed on a closed hydraulic circuit with an embedded leak system able to provide different leak apertures.

### 4.1. Leak Detection

**Figure 6:**
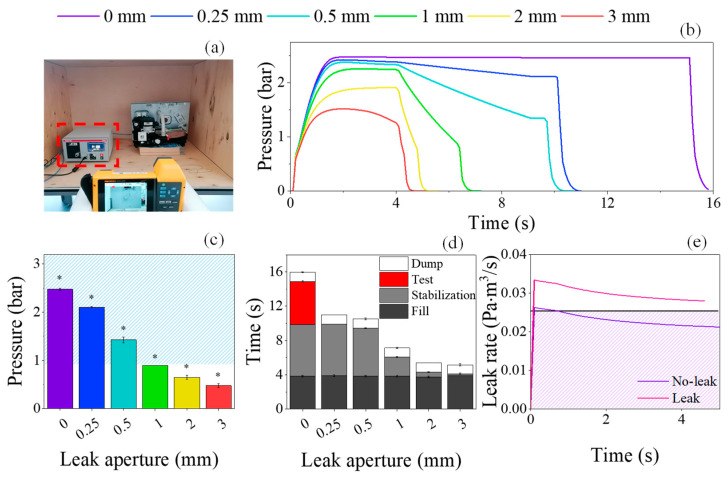
Universal leak tester ATEQ (**a**), pressure–time curves registered by the equipment (**b**), and the respective influence of leak apertures on the air pressure within the circuit (**c**). Variation of the airtightness test time with leak aperture (**d**) and the leak rate calculated during the test phase (**e**). The highlighted areas correspond to pressures higher than 0.9 bar in (**c**), and to leak rates lower than 0.025 Pa·m^3^/s in (**e**). Statistical analysis by one-way ANOVA followed by post hoc Tukey’s test: * *p* < 0.05.

Induced leaks in a hydraulic circuit were detected by the ATEQ leak tester (Figure 6a), where air pressure was highly dependent on the size of the aperture (Figure 6b). While in a non-leak scenario the pressure inside the circuit was maintained at 2.48 ± 0.02 bar, in a leak scenario the pressure was significantly reduced to 2.10 ± 0.02, 1.43 ± 0.06, 0.89 ± 0.01, 0.65 ± 0.04 and 0.48 ± 0.04 for 0.25, 0.5, 1, 2, and 3 mm leak apertures, respectively (*p* < 0.05; Figure 6c). Consequently, these variations in air pressure had a significant impact on the duration of the air tightness test. In theory, the air tightness test comprised four phases: (1) The fill phase, where the hydraulic circuit was filled with air, reaching pressures between 0.9 and 3.2 bar; (2) stabilization, during which the pressure inside the hydraulic circuit was stabilized; (3) test, where the airtightness of the hydraulic circuit was actually evaluated; and (4) dump, in which the air was expelled from the circuit.

Given that the maximum air pressure attained within the circuit using the 2 and 3 mm leak apertures was inferior to 0.9 bar, the air tightness test consisted mainly of the fill and dump phase (Figure 6d). By reducing the leak aperture from 3 to 1 mm, an increase of the overall test time was observed (from 5.18 ± 0.05 s for 3 mm to 7.15 ± 0.10 for 1 mm), which was attributed to the leak tester’s attempt to stabilize the air pressure within the circuit. For the 0.25 and 0.5 mm leak apertures, the leak tester was able to perform the stabilization phase, reaching airtightness test times of 10.95 ± 0.10 and 10.5 ± 0.18 s, respectively (Table 1). The test phase, however, was only performed for the air pressures higher than 2.4 bar, such as those of the non-leak scenario, increasing the overall test time to nearly 16 s (15.9 ± 0.12 s, Figure 6d and Table 1). During this phase, if the calculated leak rate is inferior to the threshold 0.025 Pa·m^3^/s after 5 s, it was considered a non-leak scenario (purple line, 0.02 ± 0.001 Pa·m^3^/s). Conversely, if the rate higher than 0.025 Pa·m^3^/s, it was considered a leak scenario (pink line, 0.08 ± 0.001 Pa·m^3^/s, Figure 6e and Table 1), despite attaining air pressures higher than 2.4 bar.

Whilst the equipment provided the leak rates for the case of a 0 mm aperture, it was necessary to calculate leak rates (in Pa·m^3^/s) for all leak scenarios using the air pressure and the airtightness test time (Table 1). As expected, an increasing leak rate was observed with an increased leak aperture, from 0.98 ± 0.01 Pa·m^3^/s for 0.25, to 10.55 ± 0.10 Pa·m^3^/s for 3 mm, as previously reported. Indeed, Adefila et al. verified that increasing both the leak aperture and the air pressure intensified exponentially the CO_2_ leak rate [10]. Using higher pressures (from 4 to 8 bar), Hassan et al. estimated the air flow rate from the processing of thermal images, arriving to the same conclusion [29].

### 4.2. Leak Localization

**Figure 7:**
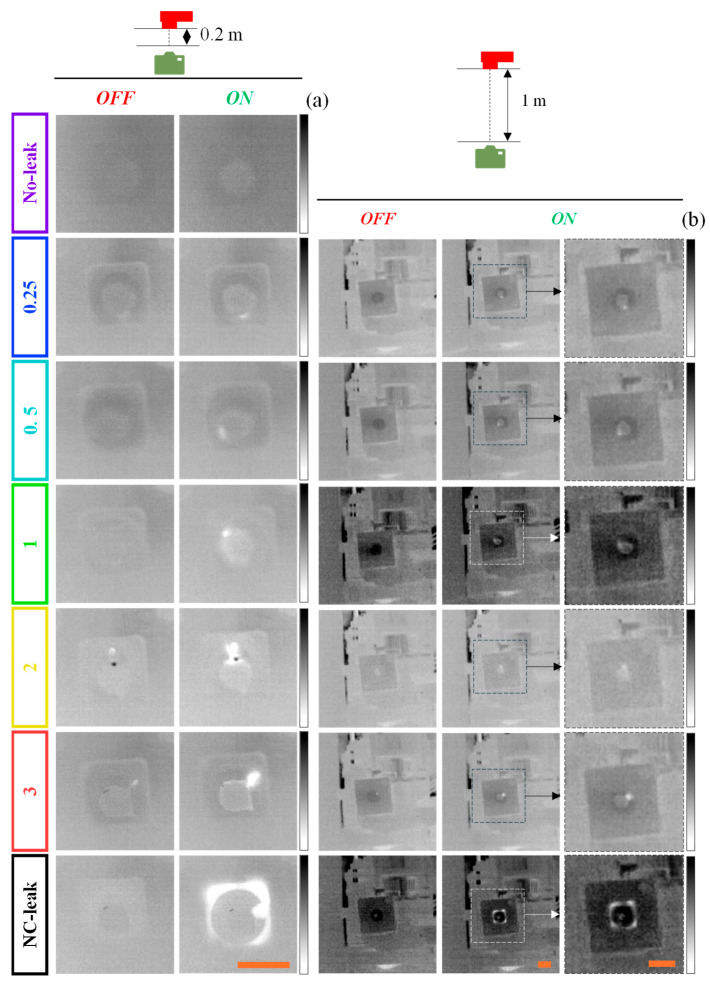
Frames of the IR videos of the leak system with different leak apertures (0, 0.25, 0.5, 1, 2, and 3 mm and NC-leak) captured at camera–leak system distances of 0.2 (**a**) and 1 m (**b**), before (air OFF) and during the airtightness test (air ON). The temperature scale ranged between 18 (white) and 24 °C (black) for all scenarios. The scale bars (in orange) indicate 20 mm. The dashed regions in (**b**) correspond to the magnified view of the leak system.

Besides the detection, the localization of air leaks is of great importance, particularly in the setting of EOL leakage stations. Passive IRT has been implemented with considerable success for the gas leak localization [30,31]; however, its performance in smaller and more intricate pipelines, such as those of closed circuits tested in EOL leakage stations, is yet to be assessed. In this regard, an IR camera was used to capture IR videos of the leak system with different leak apertures during the airtightness test in an effort to visually locate the leaks, and the resulting thermal images are depicted in Figure 7a for the camera–leak system distance of 0.2 m.

Before the airtightness test started (air OFF), there were no significant changes in pixel intensity in the thermal images associated with variation of the temperature in situ within the leak system. The small variations of color were attributed to the low emissivity of the visible portions of metallic drilled part (around 0.12–0.18 [32]), which reflected more IR radiation, increasing the pixel intensity in these small areas. Nevertheless, during the airtightness test (air ON), it was possible to visualize more pronounced white regions within the leak system triggered by the leaked air-induced local variation in temperature. Furthermore, their size and intensity were highly dependent on the leak aperture and rate, with higher leak apertures exhibiting larger white regions with higher pixel intensity than smaller leak apertures. This has been attributed to the higher temperature differences in the area surrounding the leaks with larger leak apertures, which was caused by the larger volume of leaked low-temperature compressed air [10]. Regarding the NC-leak scenario, air flow was not restricted in any leak aperture, so a larger white area was distributed through various apertures.

While these white regions were easily visible at camera–leak system distance of 0.2 m, even for the smallest leak aperture (0.25 mm), they were scarcely visible at 1 m (Figure 7b), demonstrating the influence of the distance on the performance of the IRT. In this regard, an algorithm was developed and implemented to perform leak localization.

**Figure 8:**
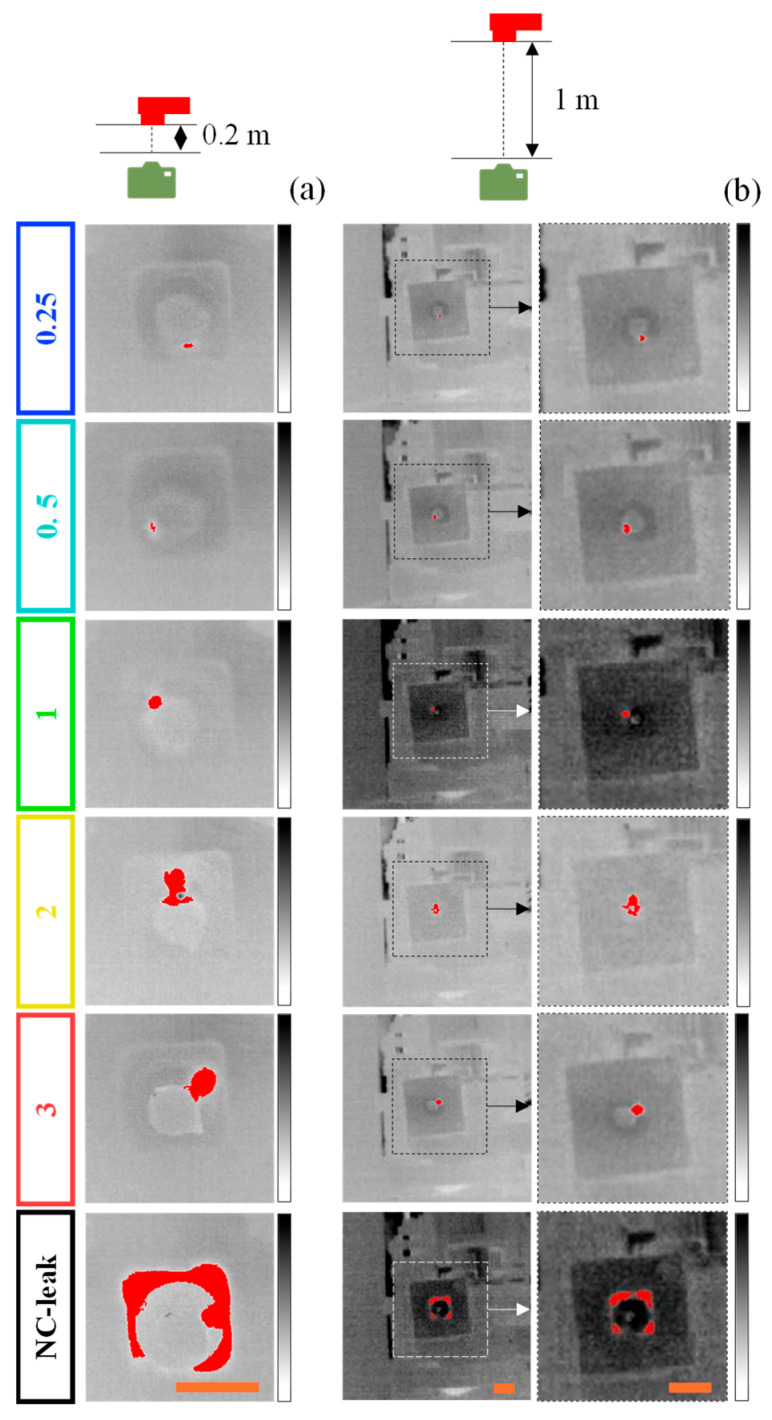
Frames of the IR videos of the air leaks with different leak apertures (0.25, 0.5, 1, 2, and 3 mm, and NC-leak) captured at camera–leak system distances of 0.2 (**a**) and 1 m (**b**) after being fed to the algorithm, which pinpointed the leak (in red) in an automated manner using a threshold of 172. The temperature scale ranged between 18 (white) and 24 °C (black) for all scenarios. The scale bars (in orange) indicate 20 mm. The dashed regions in (**b**) correspond to the magnified view of the leak system.

Figure 8a,b shows the frames of the IR videos for the camera–leak system distances of 0.2 and 1 m, respectively, with the pixels of higher intensity marked in red for an intuitive and clear indication of the leak (Video S1–S12). It should be emphasized that, despite the higher interference of lighting and temperature conditions at 1 m, particularly for the 1 mm and NC-leak scenarios, the algorithm was able to successfully locate all leaks. Indeed, the reported interference of slight variations in solar light intensity and direction on the performance of the IRT for leak localization was significantly reduced here by employing image processing techniques [23].

The value of 172 was selected as the optimal threshold for leak localization given its better overall performance, as detailed in Table 2. Indeed, for the camera–leak system distance of 0.2 m, the threshold of 172 exhibited the best accuracy (0.80 ± 0.20) and one of the best precisions (0.84 ± 0.23) and specificities (0.84 ± 0.33). Likewise, for the camera–leak system distance of 1 m, the threshold of 172 was one of the best in terms of accuracy (0.86 ± 0.11), specificity (0.98 ± 0.02), and precision (0.80 ± 0.34). Regarding recall, however, there was a slight deterioration of the performance with an increasing threshold, regardless of the distance (from 0.95 ± 0.08 for 120 to 0.74 ± 0.34 for 172 at 0.2 m and from 0.83 ± 0.27 for 120 to 0.59 ± 0.40 for 172 at 1 m). Similar simpler algorithms have been proposed for gas leak localization, typically involving thermal image processing and feature extraction for classifying leak versus non-leak scenarios. However, these approaches have not been evaluated across varying leak aperture sizes, nor have they been integrated with IRT in a comprehensive system suitable for industrial applications [27,28]. Indeed, most of these studies primarily focused on the theoretical aspects of model development, with limited assessment under real leak conditions. On the other hand, the reported advanced computer vision tools for leak detection and localization, such as machine learning or deep learning, have in general demonstrated superior performance than the localization algorithm detailed here, with reported mAP and recall values exceeding 0.95 [24,25,26]. It should be emphasized that while these tools are particularly effective in enhancing sensitivity and generalization to diverse data acquired under uncontrolled or highly variable conditions, their implementation might not only reduce the overall cost-efficiency but also hinder scalability, particularly in terms of hardware requirements and operator expertise [15]. As such, despite its lower, but acceptable, performance, the proposed algorithm was cost-effective, scalable, and compatible with the infrastructure of EOL, enhancing both its practicality and innovative value.

**Figure 9:**
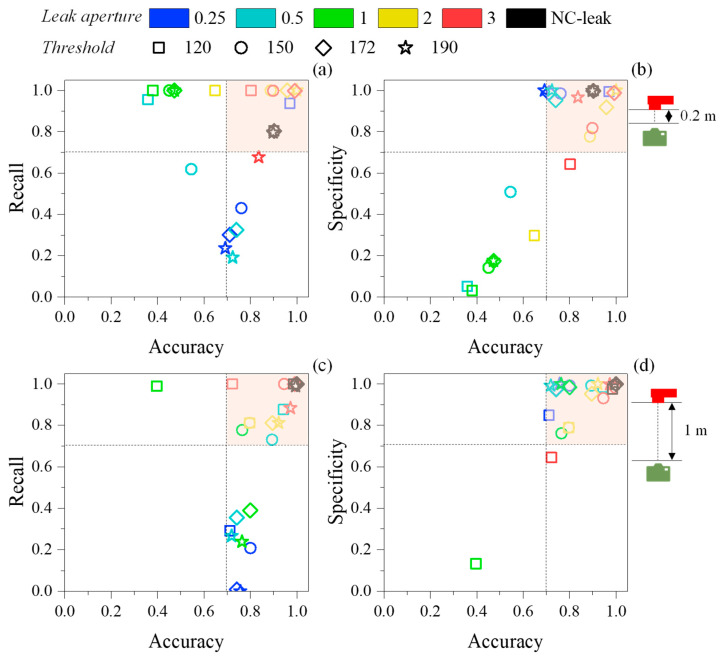
Graphs depicting the influence of the leak aperture on the algorithm’s performance metrics: accuracy versus recall for 0.2 m (**a**), accuracy versus specificity for 0.2 m (**b**), accuracy versus recall for 1 m (**c**), and accuracy versus specificity for 1 m (**d**). The highlighted area corresponds to the optimal interval (higher than 0.7) for both metrics in each graph.

Regarding the influence of the leak aperture on the algorithm’s performance, there were substantial differences between the respective metrics for each threshold, as depicted in Figure 9. For the camera–leak system distance of 0.2 m, the performance of the algorithm was considerably worse for the smaller leak apertures (0.25, 0.5, and 1 mm), regardless of the threshold (Figure 9a). In this instance, while for 0.25 and 0.5 mm the algorithm achieved a good accuracy (~0.7) and a worse recall (between 0.2 and 0.6), for 1 mm the algorithm reached maximum recall (~1) and inferior accuracy (between 0.35 and 0.5). Conversely, in the presence of larger leak apertures (2 and 3 mm and NC-leak), the algorithm achieved optimal performance (marked area in Figure 9a). Concerning specificity, most of the thresholds for most of the leak apertures were included in the interval of optimal specificity and accuracy (≥0.7), except for the thresholds for the 1 mm leak aperture. Indeed, in this case, the algorithm only achieved specificities lower than 0.2. While unusual, this phenomenon might be attributed to the lower contrast of the IR videos for this scenario that persisted despite applying the frame processing steps. It is possible that the temperature of the leak system, at this point, was slightly lower than the remaining leakage tests, affecting the algorithm’s ability to correctly identify TN.

For the camera-leak system distance of 1 m, similar trends were observed for the 3 metrics: (1) smaller (0.25, 0.5, and 1 mm) leak apertures triggered inferior recalls (<0.5) and good accuracies (between 0.7 and 0.8) (Figure 9c); (2) larger (2 and 3 mm and NC-leak) apertures promoted optimal algorithm performance in terms of both accuracy and recall (≥0.7) (Figure 9c); and (3) the IR videos of all leak apertures supported an optimal performance in terms of specificity (Figure 9d).

**Figure 10:**
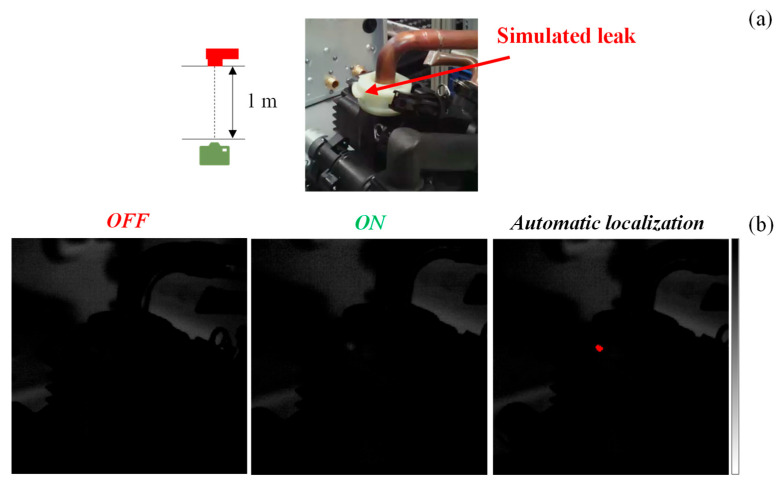
Micrograph of the hydraulic circuit (**a**) and frames of the IR videos of the hydraulic circuit in an industrial setting, captured at camera–leak system distance of 1 m before (air OFF) and during the airtightness test (air ON). These thermal videos were fed to the algorithm, which pinpointed the leak in red in an automated manner (automatic localization) (**b**). The temperature scale ranged between 19 (white) and 28 °C (black).

Overall, the developed algorithm successfully located most of the air leaks within the leak system, with accuracy higher than 0.7. In this regard, a preliminary field test was conducted in an industrial setting with non-controlled lighting conditions to validate the performance of the system. In this case, a similar hydraulic system was employed, and a leak was simulated in the location indicated in Figure 10a by disassembling components to generate a large leak. Given the non-controlled lighting conditions, it was more difficult to directly visualize the simulated leak during the airtightness test as depicted in Figure 10b (air ON). Nevertheless, after the implementation of the algorithm, the leak was clearly identified (automatic localization, Figure 10b). It should be noted that, despite needing a completely different ROI for this scenario, the algorithm was still able to correctly pinpoint the air leak with precision, demonstrating its versatility for different circuits and leak scenarios.

All in all, these results confirmed that the designed system was successful in the detection and localization of small and large air leaks in small circuits in an automated manner, in both laboratorial and industrial settings. Despite the non-controlled lighting conditions, this system was highly efficient, versatile, and cost-effective, given its simpler configuration (only an IR camera, a leak tester, and a medium-performance computer to run the algorithm) and lower computational complexity, demonstrating its potential for permanent implementation in EOL leakage stations in the manufacturing sector.

While promising so far, further optimizations can be still performed to improve its efficiency. Indeed, strategies should be exploited to increase recall, a crucial metric in the context of quality control that quantifies the percentage of true leaks that are correctly identified, including (1) reducing the camera–leak system distance and varying visualization angles to facilitate small leak visualization; (2) reducing the uncontrolled lighting interferences due to daylight variations by placing the IRT system in a dark chamber; (3) applying adaptive noise-reduction techniques to suppress background fluctuations while retaining the weaker leak-induced variations of intensity; or (4) reducing the pixel intensity threshold. Additionally, several aspects of the algorithm can be automatized to increase its versatility and reproducibility, particularly the selection of the mask for ROI definition and the choice of intensity thresholds for leak localization. For instance, by applying an automatic thresholding, mediated by the environmental lighting conditions and the properties of the materials (emissivity, for instance), leak localization can be faster and more accurate, even for smaller leak apertures. While the IR camera itself and the algorithm already provide an intuitive display with a clear indication of the leak’s location, enabling operators to efficiently identify and repair the leaks, the design of a customized user interface should be also considered in the future to further its accessibility and functionality.

## 5. Conclusions

In this work, an automatic system was designed for air leak detection and localization in EOL leakage testing stations and tested on a closed hydraulic circuit with an embedded leak system able to provide different leak apertures. This automatic system comprised (1) a universal leak tester for air leak detection; (2) an IR camera for IR video acquisition; and (3) an algorithm for frame processing and leak localization. Based on the experimental results obtained, the following conclusions can be drawn:

1. The universal leak tester successfully detected and quantified all air leaks, regardless of the leak aperture. However, its size significantly affected the air pressure retained within the hydraulic circuit, overall airtightness test time, and the resulting leak rate. In this instance, an increasing trend was observed in leak rates with increasing leak aperture.
2. The performance of the passive IRT was highly affected by the leak aperture and the camera–leak system distance. Indeed, for smaller camera–leak system distances (0.2 m), IRT allowed for effective air leak localization in closed circuits, with the size and intensity of the thermal images correlating positively with the size of the leak aperture, given the larger temperature differences triggered by the larger volumes of leaked air. However, larger camera–leak system distances (1 m) significantly impacted the performance of the IRT, particularly for smaller leak apertures.
3. The developed algorithm successfully located all air leaks in laboratorial and industrial settings, regardless of the leak aperture and the camera–leak system distance, but its performance in terms of accuracy, recall, and specificity fluctuated slightly with modifications of both these variables and the intensity threshold employed for leak localization.

All in all, these results confirmed the robustness, adaptability, simplicity, and cost-effectiveness of the developed system for air leak detection and localization, demonstrating its potential for implementation in EOL leakage stations within the manufacturing sector.

## Figures and Tables

**Table 1:** Airtightness total time and leak rate variations with leak aperture.

Leak Aperture (mm)	Airtightness Total Time (s)	Leak Rate (Pa·m^3^/s)
0 (No-leak)	15.9 ± 0.12	0.02 ± 0.001
0 (Leak)	15.2 ± 0.21	0.08 ± 0.001
0.25	10.95 ± 0.10	0.98 ± 0.01
0.5	10.5 ± 0.18	2.76 ± 0.05
1	7.15 ± 0.10	6.05 ± 0.09
2	5.38 ± 0.15	9.29 ± 0.25
3	5.18 ± 0.05	10.55 ± 0.10

**Table 2:** Recall, precision, specificity, and accuracy of the algorithm employing different thresholds.

Distance (m)	Threshold	Recall	Precision	Specificity	Accuracy
0.2	120	0.95 ± 0.08	0.66 ± 0.29	0.50 ± 0.44	0.68 ± 0.26
	150	0.81 ± 0.24	0.73 ± 0.27	0.71 ± 0.33	0.74 ± 0.20
	172	0.74 ± 0.34	0.84 ± 0.23	0.84 ± 0.33	0.80 ± 0.20
	190	0.65 ± 0.36	0.89 ± 0.24	0.86 ± 0.33	0.77 ± 0.19
1	120	0.83 ± 0.27	0.64 ± 0.29	0.73 ± 0.32	0.76 ± 0.21
	150	0.75 ± 0.29	0.83 ± 0.16	0.91 ± 0.11	0.87 ± 0.09
	172	0.59 ± 0.40	0.80 ± 0.34	0.98 ± 0.02	0.86 ± 0.11
	190	0.53 ± 0.41	0.82 ± 0.40	0.99 ± 0.01	0.85 ± 0.12

## Data Availability

Datasets generated during the current study are available from the corresponding authors on reasonable request.

## Supplementary Materials

The following supporting information can be downloaded at: https://www.mdpi.com/article/10.3390/s25113272/s1, Video S1: IR video at 0.2 m of 0.25 mm generated by the algorithm; Video S2: IR video at 0.2 m of 0.5 mm generated by the algorithm; Video S3: IR video at 0.2 m of 1 mm generated by the algorithm; Video S4: IR video at 0.2 m of 2 mm generated by the algorithm; Video S5: IR video at 0.2 m of 3 mm generated by the algorithm; Video S6: IR video at 0.2 m of NC-leak generated by the algorithm; Video S7: IR video at 1 m of 0.25 mm generated by the algorithm; Video S8: IR video at 1 m of 0.5 mm generated by the algorithm; Video S9: IR video at 1 m of 1 mm generated by the algorithm; Video S10: IR video at 1 m of 2 mm generated by the algorithm; Video S11: IR video at 1 m of 3 mm generated by the algorithm; Video S12: IR video at 1 m of NC-leak generated by the algorithm.
